# Squamous Cell Carcinoma Malignantly Transformed From Frequent Recurrence of a Presacral Epidermoid Cyst: Report of a Case

**DOI:** 10.3389/fonc.2020.00458

**Published:** 2020-04-03

**Authors:** Xiaocai Wu, Chunqiu Chen, Muqing Yang, Xiaoqi Yuan, Hong Chen, Lu Yin

**Affiliations:** Center for Difficult and Complicated Abdominal Surgery, Shanghai Tenth People's Hospital Affiliated to Tongji University, Shanghai, China

**Keywords:** presacral epidermoid cyst, squamous cell carcinoma, recurrence, malignant transformation, posterior para-sacral approach

## Abstract

**Backgroud:** Presacral tumors are rare space occupying lesions that arise in the presacral space. The incidence of presacral tumor has been reported to be 1 in 40,000 to 63,000 patients. An even rarer occurrence is the transformation of a presacral tumor into a squamous cell carcinoma (SCC).

**Case Summary:** A 61 years old man was referred to our hospital for a palpable mass near anus and appeared repeatedly in last 10 years. The patient previously underwent two surgeries at another hospital. A posterior approach was implemented in the first two surgeries, and the diagnosis was benign presacral epidermoid cyst. Two months before his admission to our department in 2017, the patient complained of a mass measuring ~2 cm around his anus. Physical examination revealed a 2 cm mass at the 12 o'clock direction in chest-knee position. A digital rectal examination indicated a rubbery lesion located in the presacral space. The Pre-operative pelvic magnetic resonance imaging (MRI) confirmed the presence of a 6.8 cm * 5.2 cm * 7.3 cm mass located at the presacral space. In contrast phase, the center of the lesion exhibited homogenous density without enhancement. The mass was then excised via posterior para-sacral approach with pathological report showing a benign epidermoid cyst after operation. The patient was discharged with full recovery without fecal incontinence. Fifteen months after being discharged from our hospital, the patient discovered a recurrence at the original site of where the mass previously appeared. Unlike the previous instance, the mass was accompanied with swelling, pain, and localized increased skin temperature. Pelvic MRI showed a 3.2 cm * 7.2 cm * 5.8 cm located at the same place, with no enhance in the core of mass. However, a speckled enhancement was observed on the margin of the lesion. The lesion was completely resected using the same procedure as before with a pathological diagnosis of SCC. The patient underwent chemoradiation therapy and remained disease free for more than 1 year.

**Conclusion:** Although very rare, benign cyst from presacral space can become malignant transformation. This highlights the importance of pre-operative diagnostic tests and evaluation to correctly identify the source of the primary cancer, which is crucial prior to starting adjuvant therapy.

## Core Tip

Presacral epidermoid cyst is considered a congenital lesion which originates from an embryologic error. They rarely become malignant transformation. In this case, we resected the tumor by posterior approach and confirmed the diagnosis of benign epidermoid cyst in the patient's third operation. The patient came back 15 months later for recurrence of the retrorectal tumor, and finally diagnosed as SCC, which was an unexpected finding. The case highlights the important of pre-operative evaluation. The malignant transformation of presacral epidermoid cyst could happen after frequent relapses.

## Introduction

In the fields of colorectal surgery, the diagnosis and discovery of retrorectal tumor in patients rarely pose much of a concern to the patients' prognosis. Tumors arising at presacral space are classified into five types (1): congenital, neuro-genic, inflammatory, osseous and miscellaneous. Approximately 60% of the congenital tumors were originated from “developmental cyst,” a concept proposed by Hawkins in 1953 (2). Developmental cysts are classified into three subtypes as epidermoid cyst, dermoid cyst and tailgut cyst. Only a few reports documenting the malignant transformation of presacral epidermoid cyst into SCC have been published (3–5). We present a case of patient initially presenting with a presacral epidermoid cyst, but later transformed into SCC.

## Case Presentation

### Chief Complaints

A 61 years old man who was referred to our hospital complained of a palpable mass around the anus and appeared repeatedly in last 10 years.

#### History of Present Disease

The patient discovered the mass around his anus by himself 10 years ago. He underwent two major surgeries for resection of the mass by posterior approach. A palpable lesion appeared 2 months ago prior to admission into our hospital.

#### History of Past Disease

The patient was diagnosed with hypertension and took single dose of calcium channel blockers daily. His blood pressure was under control and well-managed.

#### Physical Examination

A 2 cm mass was observed at the 12 o'clock direction in chest-knee position. Digital rectal examination determined the presence of a rubbery lesion located in presacral space. The mass was smooth and firm, and no nodule was felt on the surface. The mass was tender and the local skin temperature was slightly elevated.

#### Laboratory Examination

Cell blood count revealed mild anemia with a hemoglobin count of 122 g/L along with normal white blood cell and platelet count. The blood biochemistries, hematological tumor markers, prothrombin, and partial thromboplastin were normal. Electrocardiogram, chest computed tomography, echocardiography and pulmonary function tests were also normal.

#### Imaging Examination

**Figure 1:**
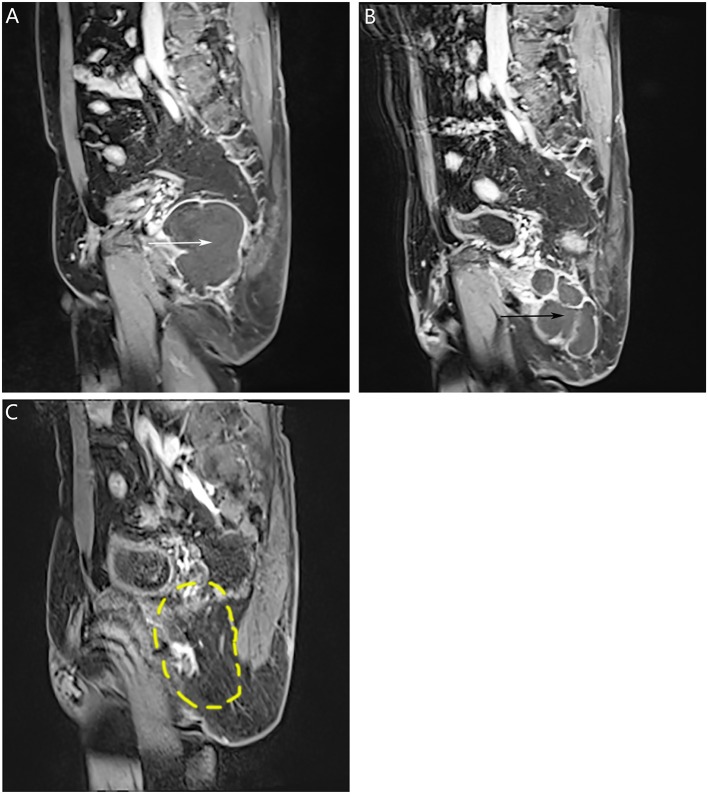
Pre-operative pelvic MRI. **(A)** Before first surgery in our hospital, contrast-enhanced pelvic MRI in sagittal view showed a 6.8 cm * 5.2 cm * 7.3 cm mass located at presacral space (white arrow). **(B)** Before second surgery in our hospital, contrast-enhanced pelvic MRI in sagittal view showed a 3.2 cm * 7.2 cm * 5.8 cm mass located at presacral space (black arrow). **(C)** One year after final surgery, enhanced pelvic MRI showed no signs of recurrence (the yellow dotted circle indicated the location of the previous mass).

Before the first surgery in our hospital, pre-operative pelvic magnetic resonance imaging (MRI) with intravenous Gadolinium highlighted a 6.8 cm * 5.2 cm * 7.3 cm mass located at presacral space. In contrast phase, the center of the lesion showed homogenous density and without enhancement (Figure 1A, white arrow). Before second surgery in our department, MRI showed a 3.2 cm * 7.2 cm * 5.8 cm mass located at presacral space (Figure 1B, black arrow). No enlargement of lymph nodes was observed. After gadolinium administration, the center of the lesion showed homogenous density without enhancement. This time however, we observed a speckled pattern enhancement on the margin surrounding the lesion. Due to the MRI finding, our differential diagnosis included presacral epidermoid cyst relapse, without exclusion of malignant tumor.

### Treatment

The treatment approach was surgical resection of the entire tumor via posterior para-sacral approach. A long star retractor was used to retract and expose the lesion. A negative pressure drainage tube was placed and removed on the seventh day post-surgery. The patient was discharged 1 week after surgery with full recovery and no fecal incontinence.

### Outcome and Follow-Up (Including Pathological Diagnosis)

**Figure 2:**
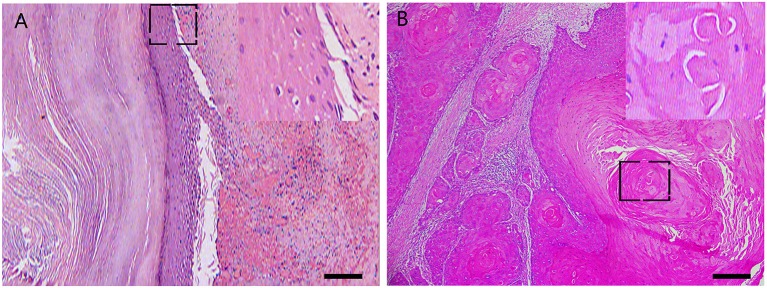
Histological features of the resected tumors. Representative pathological photos of the third surgery: **(A)** original magnification × 100 and insert × 400. Representative photos of the latest surgery: **(B)** original magnification × 100 and insert × 400.

Pathological diagnosis of the resected tumor was benign presacral epidermoid cyst in the third surgery (Figure 2A, original magnification ×100 and insert ×400). Pathological finding in the latest surgery: a mass measured 8 * 5 * 3 cm, with the appearance of saclike, white to tan and necrotic. The final diagnosis was SCC (Figure 2B, original magnification ×100 and insert ×400). The patient completed standard pelvic radiation and 12 courses of chemotherapy. One year after his final surgery, enhanced pelvic MRI showed no signs of tumor recurrence (Figure 1C, the yellow dotted circle indicated the location of the previous mass). The patient was advised to come for regular follow-up visits for once a year during the first three-year post-surgery.

## Discussion

The presacral epidermoid cyst is a rarely occurring condition, the majority of them presenting without any clinical symptoms. Most of these cysts are found incidentally on radiological imaging for other purpose, while a few portions present to doctors with compression of adjacent tissues thereby producing symptoms of discomfort. Common presenting symptoms include: constipation, urinary retention and a palpable mass near anus.

This case highlights the repeated recurrence of an epidermoid cyst for a duration of 10 years. The typical treatment approach for benign epidermoid cyst involves an entire surgical resection of the tumor. Tumor recurrence rarely develops except for those that have been incompletely resected (6–8). Our patient underwent four major surgeries in last 10 years. The first three of them were benign epidermoid cyst, which suggests an incompletely excision of the tumors during the first two surgeries. However, the diagnosis of SCC was established after last surgery, which serves to remind us that tumor recurrence is a major characteristic possessed by malignant tumor and that it should serve as an important clue for differential diagnosis when ordering diagnostic tests for a patient.

Presacral tumors are generally managed by either colorectal surgeons or orthopedists. Surgeries performed by these specialists generally incorporate a trans-abdominal, trans-sacral and combined abdominal-sacral approach, whereas fewer surgeons chose trans-rectal (6) and trans-sphincteric (9) approach instead. The trans-vaginal approach is less mentioned in literature due to the fact that only a small number of patients were treated by gynecologists, who think that trans-vaginal approach has the merit of less operative time, less blood loss and should not be neglected (10, 11). Successful reconstruction after en-bloc resection of large tumor in pelvis is challenging due to a large soft tissue defect (12), so Patrick B and his associates conducted a retrospective review and suggested a combination of gluteus maximus (GLM) flaps and human acellular dermal matrix (HADM) to reconstruct bony and soft tissue defection. With trans-sacral approach, a rotation flap is not needed because there is no tension in suturing once this patient's tumor is excised. No infection or other wound healing problems was observed after surgeries.

In this patient's case, the surgical team did not realize that the patient's previously diagnosed presacral epidermoid cyst had transformed into a malignant SCC at the time of patient discharge from the hospital. The patient was referred to the oncology department because the tumor ruptured during the surgery. To prevent cancer recurrence, concomitant external beam radiation (with a total dose of 60 Gy and delivered in 25 fractions of 2.4 Gy during 5 weeks period) and adjuvant chemotherapy were implemented. The chemotherapy regimen of gemcitabine plus cis-platinum was given, which is standard protocol for lung SCC (13). The BEP regimen (bleomycin, etoposide, cisplatin) is considered to be the standard first line chemotherapy (14, 15) for germ cell cancers whereby the transformation of an epidermoid cyst into a SCC can be considered one as such. The patient completed 12 chemotherapy regimens of gemcitabine plus cis-platinum. The value of post-surgery chemotherapy for malignant germ cell cancer is still controversial (16). However, 12 courses of chemotherapy are very taxing on the patient's overall health, for post-surgery chemotherapy of intermediate-risk (IR) malignant germ cell tumors, four cycles of BEP is standard regimen (17). Although the patient remained disease-free for more than 1 year (Figure 1C, the yellow dotted circle indicated the location of the previous mass), this case highlights the importance of teamwork in medical care to deliver optimal patient treatment.

## Conclusion

Although it is very rare, benign cyst from the presacral space can become malignant transformation. This case highlights the importance of pre-operative diagnostic tests and evaluation to correctly identify the source of the primary cancer, which is crucial prior to starting adjuvant therapy.

## Data Availability Statement

All datasets generated for this study are included in the article/supplementary material.

## Ethics Statement

The studies involving human participants were reviewed and approved by ethics committee of Shanghai Tenth People's Hospital Affiliated to Tongji University. The patients/participants provided their written informed consent to participate in this study. Written informed consent was obtained from the individual(s) for the publication of any potentially identifiable images or data included in this article.

## Author Contributions

XW reviewed the literature and drafted the manuscript. LY performed the surgeries and made important revisions to the manuscript. CC contributed to drafting the manuscript. XW, MY, XY, HC, and CC assisted during the surgeries. All authors have approved the submission of this manuscript.

### Conflict of Interest

The authors declare that the research was conducted in the absence of any commercial or financial relationships that could be construed as a potential conflict of interest.
